# Reticulate evolution in stick insects: the case of *Clonopsis *(Insecta Phasmida)

**DOI:** 10.1186/1471-2148-10-258

**Published:** 2010-08-25

**Authors:** Liliana Milani, Fabrizio Ghiselli, Marco Pellecchia, Valerio Scali, Marco Passamonti

**Affiliations:** 1 Dipartimento di Biologia Evoluzionistica Sperimentale, University of Bologna, Bologna, Italy; 2 Istituto di Zootecnica, Catholic University of Sacred Heart, Piacenza, Italy

## Abstract

**Background:**

Phasmids show noteworthy abilities to overcome species-specific reproductive isolation mechanisms, including hybridization, polyploidy, parthenogenesis, hybridogenesis and androgenesis. From an evolutionary standpoint, such tangled reproductive interactions lead to the complex phyletic relationships known as "reticulate evolution". Moroccan stick insects of the genus *Clonopsis *include one bisexual (*C. felicitatis*) and two closely related parthenogenetic forms (*C. gallica*, *C. soumiae*), which represent a polyploid series in chromosome number, but with apparent diploid karyotypes. Moreover, two *Clonopsis *strains of ameiotic males have been described, *C. androgenes-35 *and *C. androgenes-53*. As a consequence, *Clonopsis *stick insects may have experienced complex micro-evolutionary events, which we try to disentangle in this study.

**Results:**

Mitochondrial *cox2 *analysis supports a recent divergence of *Clonopsis*, while AFLPs evidence genetic differentiation not linked to karyotypes, so that parthenogenetic *C. gallica *and *C. soumiae *appear to be a mix of strains of polyphyletic origin rather than single parthenogenetic species. Moreover, an admixed hybrid origin seems to be confirmed for *C. androgenes*.

**Conclusion:**

On the whole, *Clonopsis *is an intriguing case of reticulate evolution. Actually, complex cladogenetic events should be taken into account to explain the observed genetic structure, including diploidization of polyploid karyotypes, possibly coupled with hybridization and androgenesis. We also proposed a "working hypothesis" to account for the observed data, which deserves further studies, but fits the observed data very well.

## Background

Gene duplication, by which identical copies of genes are created within a single genome, is a major evolutionary process in producing new functions in eukaryotes. Among the possible mechanisms to gain extra copies of genes, whole genome duplication has been suggested as an important step in the production of evolutionary novelties, such as in Vertebrates, *Arabidopsis *and other eukaryotes (see [1,2] for reviews). In such lineages, ancient polyploidization events have been suggested, followed by a process of diploidization of the genome (i.e. the evolutionary process whereby a polyploid species 'decays' to become diploid; [1]). Polyploidy is a common feature of stick insects (Phasmida), so they might be a system of choice to study many aspects of polyploidization. Phasmids also show a noteworthy ability to overcome species-specific reproductive isolation mechanisms, so that hybrids are often found [3]. Such hybrids may show high levels of heterozigosity, which, particularly when co-occurring with polyploidy, may allow them to persist for a long time (i.e. their allelic richness would permit better adaptation to changing environments). Hybridization leads also to parthenogenesis, and parthenogenetic females may not be completely isolated from the closest taxa, since backcrosses to paternal or related species may occur to incorporate additional genomes, thus increasing ploidy level and overall genetic variability [4-6]. From an evolutionary standpoint, such tangled reproductive interactions lead to the complex phyletic relationships known as "reticulate evolution" [3].

To make reticulate evolution of phasmids even more complicated, stick insects may also reproduce by androgenesis, as first discovered in *Bacillus *hybrids [7] and recently proposed to account for *Leptynia attenuata *fast chromosome evolution, as well as for the origin of *Pijnackeria hispanica *(formerly *Leptynia*) parthenogens [4,6,8].

Another intriguing cytological feature of Phasmida is their extreme karyotype diversity and their capability for deep chromosomal repatterning [8-11]. Moreover, in parthenogenetic forms the chromosome number is extremely variable, and up to 100 can be found [12].

This paper deals with the molecular characterization of the stick insect genus *Clonopsis*, focusing on North African strains. The genus included two bisexual North African species, *Clonopsis maroccana *Bullini & Nascetti (*cn *= 22/21, XX/X0) and *Clonopsis algerica *Pantel (*cn *= 32/31, XX/X0), and an obligate parthenogenetic species, *Clonopsis gallica *Charpentier, with a wide distribution in North Africa and Europe, showing a karyotype of 54 - 57 chromosomes. Recent studies on Moroccan samples revealed the existence of two additional North African taxa related to *C. gallica*: the bisexual *Clonopsis felicitatis *Scali & Milani (*cn *= 36/35, XX/X0) and the all-female *Clonopsis soumiae *Scali & Milani (*cn *= 72) [13]. Together with *C. gallica*, they form a numerically polyploid series with haploid number *n *= 18, being *C. felicitatis *diploid (*cn *= 36), *C. gallica *triploid (*cn *= 54) and *C. soumiae *tetraploid (*cn *= 72). Quite surprisingly however, they all seem to be diploid by standard karyotyping techniques (see [5] for detailed pictures of the karyotypes), so diploidization was considered for these forms, like for the sexual and parthenogenetic stick insect *Sipylodea nelida *[5,10]. Furthermore, two strains of ameiotic males, *C. androgenes-35 *and *C. androgenes-53*, with *cn *= 35 (X0) or 53 (X0) diploid karyotypes were found, which, it is suggested, maintain themselves as clonal androgens: these are thought to derive from diploid species hybrids, which, by skipping meiosis, may produce unreduced but genetically and chromosomally balanced sperm, thus becoming able to father clonal sons when inseminating the eggs of the syntopic all-female *C. soumiae *by excluding its genetic contribution [5].

With such a complex array of unusual reproduction modes, the genus *Clonopsis *represents an intriguing system to study reticulate evolution. Since the whole genome sequence of those insects is not available at the moment, we used a more 'classic' approach to their characterization. Previous papers on phasmids showed that the combined use of nuclear and mitochondrial markers may give significant results, therefore, to understand *Clonopsis *micro-evolution we used the mitochondrial cytochrome oxidase subunit 2 (*cox2*) and AFLP markers. The *cox2 *gene has been sequenced over a wide variety of phasmid taxa and has proved useful for phylogenetic research [4,8,14], while AFLPs, since their introduction in 1995 [15], have been successfully used on a large number of organisms, bringing key answers to major biological issues [16], and have been used to characterize the stick insect genus *Timema *[17]. As a matter of fact, studies have demonstrated that AFLPs have the capacity to resolve extremely small genetic differences [18,19], so they have been proposed as the best markers to show up population genetic variability, when compared to other available molecular approaches, such as microsatellites, multigene DNA sequencing and SNPs [20]. AFLPs have also proved to be a very powerful tool for the identification of interspecific and intraspecific hybrids [21], even in systems where microsatellites have failed to do so [22]. For all the above mentioned reasons, AFLPs seem to be a good choice for unraveling *Clonopsis *population structure and to obtain data that might eventually shed light on the role of diploidization in their evolution.

Our data suggest that parthenogenetic *C. gallica *and *C. soumiae *are a mix of strains of polyphyletic origin rather than single parthenogenetic species, all of them supposedly diploid. To account for this, we discuss *Clonopsis *reticulate evolution in the light of the known mechanisms of karyotype diploidization. Finally, we propose a testable hypothesis for *Clonopsis *micro-evolution, which has the merit of fitting in with our data. Of course, our scenario for *Clonopsis *deserves more in-depth analyses to be tried, nevertheless we feel that this is a sound "working hypothesis".

## Results

### Pertinent information on analyzed samples are reported in Table 1 and Figure 1

**Table 1:** Collecting sites and acronyms of analyzed *Clonopsis *specimens.

COLLECTING SITES	SPECIES	SPECIMENS *cox2*	**Acc. No**. GenBank	SPECIMENS AFLP
				
**MOROCCO**				
				
Tetouan (TET)	*C. felicitatis *(*cn *= 36/35, XX/X0)	mTET8	GQ370547	mTET8
		mTET22	GQ370548	mTET22
		mTET38	GQ370549	-
		mTET39	GQ370550	mTET39
		mTET55	GQ370552	mTET55
		mTET56	GQ370553	mTET56
		mTET57	GQ370554	mTET57
		fTET21	GQ370542	fTET21
		fTET24	GQ370551	fTET24
		fTET36	GQ370578	-
		fTET37	GQ370543	-
				
Taferiate (TAF)	*C. gallica *(*cn *= 54)	fTAF3	GQ370574	fTAF3
		fTAF4	GQ370555	fTAF4
				
Oued Laou Area (OLA)	*C. gallica *(*cn *= 54)	fOLA1	GQ370572	fOLA1
		-	-	fOLA12
		fOLA27	GQ370536	fOLA27
				
Chefchaouen (CHA)	*C. soumiae *(*cn *= 72)	fCHA26	GQ370529	fCHA26
				
Sefliane (SEF)	*C. soumiae *(*cn *= 72)	fSEF7	GQ370537	fSEF7
		fSEF28	GQ370538	-
		fSEF42	GQ370541	fSEF42
		fSEF54	GQ370573	fSEF54
	*C. gallica *(*cn *= 54)	fSEF40	GQ370539	fSEF40
		fSEF41	GQ370540	fSEF41
				
Targuist (TAR)	*C. soumiae *(*cn *= 72)	-	-	fTAR48
		fTAR49	GQ370558	fTAR49
		fTAR50	GQ370576	fTAR50
		fTAR64	GQ370577	fTAR64
	*C. androgenes-35 *(*cn *= 35)	mTAR5	GQ370544	mTAR5
	*C. androgenes-53 *(*cn *= 53)	mTAR43	GQ370545	mTAR43
	*C. androgenes-53 *(*cn *= 53)	mTAR47	GQ370546	mTAR47
	*C. maroccana *(*cn *= 22/21, XX/X0)	fTAR6	GQ370575	fTAR6
				
**SPAIN**				
				
Benissa (GBE)	*C. gallica *(*cn *= 54-57)	fGBE197	GQ370530	-
		fGBE293	GQ370531	-
		fGBE297	GQ370532	-
				
El Bosque (GEL)	*C. gallica *(*cn *= 54-57)	fGEL1	GQ370533	-
		fGEL2	GQ370559	-
		fGEL3	GQ370560	-
				
Espuña (GEP)	*C. gallica *(*cn *= 54-57)	fGEP529	GQ370561	-
				
Laujaon (GLA)	*C. gallica *(*cn *= 54-57)	fGLA198	GQ370566	-
		fGLA317	GQ370567	-
		fGLA320	GQ370534	-
				
Paterna del Rio (GPR)	*C. gallica *(*cn *= 54-57)	fGPR304	GQ370565	-
				
San Pedro Alcantara (GSP)	*C. gallica *(*cn *= 54-57)	fGSP577	GQ370535	-
				
**PORTUGAL**				
				
Monchique (GMO)	*C. gallica *(*cn *= 54-57)	fGMO312	GQ370562	-
		fGMO313	GQ370563	-
		fGMO314	GQ370564	-
				
Portalegre (GPO)	*C. gallica *(*cn *= 54-57)	fGPO530	GQ370579	-
				
**ITALY**				
				
Bottinaccio (GBO)	*C. gallica *(*cn *= 54-57)	fGBO337	GQ370556	-
				
Rio Torto (GRT)	*C. gallica *(*cn *= 54-57)	fGRT526	GQ370568	-
		fGRT527	GQ370569	-
				
Villadoria (GVI)	*C. gallica *(*cn *= 54-57)	fGVI1	GQ370570	-
		fGVI2	GQ370557	-
		fGVI3	GQ370571	-
				
**Outgroups**	*Bacillus grandii grandii*	-	AF038220.2	-
	*Bacillus atticus atticus*	-	AF148316.1	-
	*Bacillus rossius redtenbacheri*	-	AF038205.2	-

Acronyms and number of the specimens according to [5].

**Figure 1:**
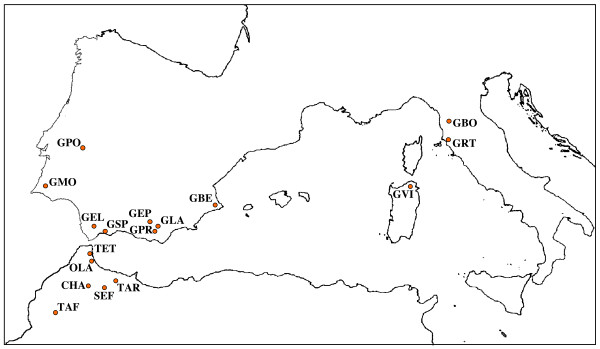
**Moroccan and European collecting sites with acronyms of *Clonopsis *specimens**. Acronyms: Italy: GBO = Bottinaccio, GRT = Riotorto, GVI = Villadoria. Spain: GBE = Benissa, GEL = El Bosque, GEP = Espuña, GLA = Laujaon, GPR = Paterna del Rio, GSP = San Pedro Alcantara. Portugal: GMO = Monchique, GPO = Portalegre. Africa (Morocco): CHA = Chefchaouen, OLA = Oued Laou, SEF = Sefliane, TAF = Taferiate, TAR = Targuist, TET = Tetouan.

**Figure 2:**
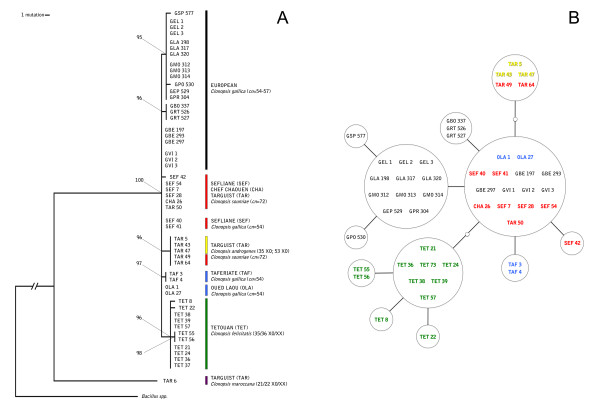
***cox2 *analyses**. (A) Bayesian analysis performed using MrBayes 3.1 (20,000,000 generations; [49]). (B) Templeton's network [50] obtained with TCS 1.21 [51]. *Bacillus grandii grandii*, *Bacillus atticus atticus *and *Bacillus rossius redtenbacheri *were utilized as outgroups in phylogenetic reconstructions based on *cox2*. Acronyms as in Table 1.

The *cox2 *alignment showed very low variability among all analyzed *Clonopsis *specimens, except for *C. maroccana*, which clearly splits away from the cluster that includes *C. felicitatis*, *C. gallica *and *C. soumiae*. Consequently the latter three species are more closely related and might be considered as a species complex. However, it is worth noting that *cox2 *haplotype distribution appears to show little relationship with karyotypes; the Bayesian tree (Figure 2A) showed up a few significant groups (i.e. *pp *≥ 95): *i) *two clusters including most, but not all, European *C. gallica *(*cn *= 54); *ii) *a cluster which quite surprisingly includes most of the specimens from Targuist, regardless of their chromosome number (TAR; *cn *= 35, 53, 72); *iii*) a small cluster including the two females from Taferiate (TAF; *cn *= 54); *iv) *a cluster including all amphygonic *C. felicitatis *from Tetouan (TET; *cn *= 36/35, XX/X0); finally, a large unresolved polytomy, including other European and North African *C. gallica *(*cn *= 54), as well as *C. soumiae *(*cn *= 72) specimens (see Figure 1 for population acronyms). Although some phylogenetic signal is present, the *cox2*-based phylogeny does not appear to resolve the relationships between the above-mentioned groups, mainly because of the very low level of overall variability; moreover the parthenogenetic taxa *C. gallica *and *C. soumiae *might be polyphyletic.

To better unravel haplotype relationships in such a low variability framework, we applied the Templeton's network, which has already proved useful in such situations ([4] and references therein). The network (Figure 2B) showed that: *i) *all amphygonic specimens from Tetouan (TET; *C. felicitatis*) cluster together and show only a little variability (green); *ii) *as above, two separate clusters include most, but not all, European *C. gallica *(black); *iii) *all but one (TAR50) specimens from Targuist are still joined and they all share the same haplotype, regardless of their karyotype; *iv) *the rest of the haplotypes form a quite homogeneous group, with *C. gallica *and *C. soumiae *mixed together, and with only TAF3, TAF4 and SEF42 being different for one substitution.

For AFLPs, the three used highly polymorphic primer combinations (see 'Methods') detected a total of 195 markers for the 27 analyzed individuals. Compared to *cox2*, AFLP markers show a higher level of variability and enable better differentiation among *Clonopsis *populations.

**Figure 3:**
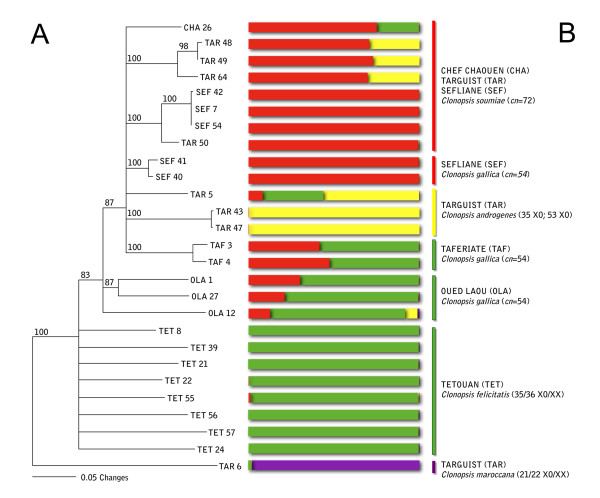
**AFLP analyses**. (A) AFLP-based Minimum Evolution tree calculated using PAUP 4.0 [56]; support for each node was obtained using bootstrap (1000 replicates, [57]). *C. maroccana *has been used as outgroup. (B) Genetic structure, as obtained by Bayesian analysis (STRUCTURE). A probability of membership *q *≥ 80% was chosen to consider a genotype assigned to one population, while a probability under the threshold means that the individual may have different parental populations, and consequently is admixed via hybridization. Acronyms as in Table 1.

The AFLP-based Minimum Evolution tree (ME, Figure 3A) showed up a basal polytomy for the bisexual *C. felicitatis *(TET), while all unisexuals separate with quite a significant bootstrap value. Within unisexuals, some *C. gallica *appear to be basal (i.e. OLA), while some others (TAF, SEF) are joined in a large polytomy with *C. soumiae*, and the androgenetic males TAR5 (*cn *= 35), TAR43 and TAR47 (*cn *= 53).

The genetic structure obtained by Bayesian analysis (STRUCTURE software) gave additional clues regarding the genetic diversification in *Clonopsis*. The graph is reported on Figure 3B, aligned to the ME tree for easier comparison. Five replicates for each value of K (from 1 to 12) were run under the admixture model with a burn-in period of 50,000 and 750,000 iterations. The admixture model means that individuals may have mixed ancestry. Moreover, this model appears very useful for stick insects, as it can deal with hybrid zones in a natural way. No prior information was used, and all individuals were deemed as diploid, as shown up by karyotypes. A probability of membership *q *≥ 80% was chosen to consider a genotype assigned to one population, while a lower-than-threshold probability means that the individual may have different parental populations, and consequently is admixed via hybridization. In order to determine K, the smallest value of the estimated probability of data was chosen, and this value was then compared with the more formal method proposed by [23]. Both methods gave 4 as the most probable value of K, so we can assume that the dataset includes 4 parental populations that are distinguishable on a genetic basis. One includes the single specimen of *C. maroccana *(TAR6; purple); a second parental population includes the amphigonic individuals from Tetouan (TET, green); a third one the parthenogenetic females from Sefliane (SEF) and a female from Targuist (TAR50) (red), and a fourth one the two males with *cn *= 53 (TAR43, TAR47; yellow). All other individuals are not assigned with certainty, and therefore are considered of hybrid origin from between two or more parental populations.

It should be noted that the groupings obtained with the Bayesian analysis mirror the results of the Factorial Correspondence Analysis (FCA; see additional file 1); moreover FCA showed that two groups are the most divergent among the analyzed samples (i.e. TET bisexuals and TAR/SEF females). For this reason, we proceeded in our analysis by forcing the software to accept K = 2. Using this sub-optimal setting, the software brings together individuals in two populations only, which are formed by the elements that are most genetically pure and different, and therefore better indicating specimens with admixed genome. Actually, 10 more runs with K = 2 (burn-in 50,000; 100,000 iterations; admixture model, no prior information) were made without *C. maroccana *(its presence "pushes" the other individuals to the top of the chart, as it assumes an outgroup behavior; see additional file 1). The two parental populations identified with this analysis (data not shown) include on one side the amphigonic *C. felicitatis*, on the other side the parthenogenetic females from Sefliane (both *C. soumiae *and *C. gallica*) and Targuist (*C. soumiae*); both were assigned to one of the two parental populations with a probability equal or close to 100%. In addition, two of the three parthenogenetic *C. gallica *from Oued Laou were assigned to the geographically close population of Tetouan (the diploid *C. felicitatis*, with a probability of just over 80%), while the remaining one (OLA1) is shown to have a hybrid genotype. All the androgenetic males (both *cn *= 53 and *cn *= 35) show an admixed genome. Moreover, two *C. gallica *specimens (TAF3 and TAF4) as well as a single *C. soumiae *(CHA26) are indeed admixed too.

On the whole, the pattern of admixture is quite puzzling: although most of the specimens do not show up hybrid genomes, in both *C. gallica *and *C. soumiae *we found specimens with statistically-supported admixed genomes. Moreover, *C. androgenes *males are found, indeed, to have admixed genomes at least with K = 2.

## Discussion

To disentangle *Clonopsis *evolution, we proceeded with mitochondrial and nuclear DNA analysis, with the aim of finding traces of possible hybridization/polyploidization events, as was the case with other analyzed stick insects ([4] and references therein). Mitochondrial *cox2 *gene analysis showed extremely low haplotype variability, except for *C. maroccana*, which was very different from all other sampled *Clonopsis*, in line with its highly differentiated karyotype. The mtDNA data for the remaining *Clonopsis *seemed to support a very recent divergence, as they did not allow splitting of the two parthenogens *C. gallica *and *C. soumiae*. On the other hand, AFLP markers showed a good genetic differentiation and distinct groups were detected (K = 4; Figure 3B): one is *C. maroccana *(purple color), thus confirming mtDNA data; another group is the bisexual *C. felicitatis *(green) which appears to be a well-defined taxon; a third group (red) is formed by specimens of both *C. gallica *(*cn *= 54) and *C. soumiae *(*cn *= 72), with no relation to the different chromosome number. The other *C. gallica *and *C. soumiae *specimens showed quite a variable genetic constitution, with either admixed or non-admixed genotypes. On the whole, *C. gallica *and *C. soumiae *parthenogens did not appear to be homogeneous taxa, but rather a mix of strains of polyphyletic origin, either hybrids or not. Moreover, an admixed hybrid origin seemed to be confirmed for *C. androgenes*: the male with *cn *= 35 is invariably admixed, while a hybrid origin of *cn *= 53 males is supported only for K = 2 (data not shown). This is strong evidence that complex cladogenetic events could account for the observed *Clonopsis *genetic structure.

When trying to describe *Clonopsis *evolution, there is a preliminary observation to make: as already mentioned, *C. felicitatis*, *C. gallica *and *C. soumiae *seem to be a reasonable polyploid series with *n *= 18, i.e. they all have karyotypes that are exact multiples of 18 chromosomes (36 for *C. felicitatis*, 54 for *C. gallica *and 72 for *C. soumiae*). This would be hard to explain without hypothesizing that polyploidization events originated parthenogenetic *C. gallica *and *C. soumiae *strains, since it would be extremely odd to imagine that parthenogenetic forms, with karyotypes that are the exact multiple of the closest bisexual species, could have formed other than through polyploidy. Moreover, in stick insects the haploid chromosome number generally ranges from 16 to 20 [24]. For the above mentioned reasons, we feel confident in assuming that polyploidization might have had a major role in *Clonopsis *evolution, as in that of many other stick insects.

Increases in ploidy level can be achieved by means of several mechanisms during gametogenesis (i.e. pre-meiotic, intra-meiotic or post-meiotic restitution). Invariably, the outcome of these mechanisms is the production of diploid gametes, which, after fertilization, would increase the ploidy of the progeny. Such mechanisms may also include some meiosis (automixis) or not (apomixis), and have already been experimentally confirmed in several complexes [25]. In any case, whenever these mechanisms were confirmed, a clear polyploid karyotype would be recovered.

However, as mentioned before, *C. gallica *and *C. soumiae *look morphologically diploid on careful analysis of their karyotypes: actually, although most chromosomes are quite similar, at least some of the biggest cannot be arranged in triplets or quadruplets (see [5] for detailed pictures of *Clonopsis *karyotypes). As a consequence, if we accept that both *C. gallica *and *C. soumiae *strains are polyploids with diploid karyotypes, then they must have experienced large genome restructuring events, leading to diploidization.

Diploidization of polyploid genomes has been observed in autopolyploids and, most commonly, in allopolyploids. However, despite the increasing evidence of polyploidization as a major force in evolution, the molecular basis of diploidization is far from being fully understood, although many mechanisms have been suggested to date. For instance, tetraploid salmonids are the best-documented case of animal diploidization in autopolyploids: the process proposed took a long time and seems to be still in progress, since it is suggested that it started when, or shortly after, the family originated about 25-100 Mya [26]. This certainly seems not to be the case of *Clonopsis*, because its low mtDNA variability appears to be an indication of a recent origin of the parthenogenetic strains; therefore, a much quicker diploidization process has to be hypothesized.

Diploidization has been commonly related to hybridization events, followed by a massive rearrangement of the genome [27]. The process, as summarized in [28], might be as follows: allopolyploids may arise by hybridization of two individuals whose chromosomes (the so-called homoeologues) have similar gene order, but differ in repetitive DNA content (such as in closely-related species or races); after hybridization, homoeologues can go through the loss or gain of genes and repetitive sequences, as well as structural chromosome translocations, thus increasing their morphological divergence. Actually, the extent of structural rearrangements and sequence elimination may vary: the more extensive the rearrangements are, the more distinct the chromosomes will be, so that if the distinctions become extensive enough the species will effectively gain a diploid set.

In more detail, data from [29] on non-coding sequences suggest a faster way to diploidization, in which a quick DNA elimination, just after polyploidization, takes place: they found that specific sequences named CSSs (Chromosome-Specific Sequences) and GSSs (Genome-Specific Sequences) are eliminated, and suggested a regulatory role for such sequences in the physical chromosome behavior. This leads to the conclusion that the appearance of new polyploid species may be followed by deep genomic changes in a short time, and polyploidization may be an accelerating evolutionary factor itself [29]; indeed, the finding that one genome can eliminate up to 14% of its loci in a single generation shows that allopolyploidy may lead to the establishment of a new species in one step, and that large-scale genomic rearrangements can occur very quickly [30].

Might this be the case with *Clonopsis*? Compared to autopolyploidization, allopolyploidization is a revolutionary event generating two genomic "shocks" in the newly-formed individual, namely hybridity and polyploidy: the former implies that two different genomes are brought together in the same nucleus, the latter results in duplicated genomes. Consequently, the genomes undergo a series of irreversible reorganizations, like structural re-patterning of chromosomes [31], changes at sequence level, regulation of gene expression, activation of transposons [32], and amplification, re-assortment or elimination of highly repetitive sequences and low copy sequences [33]. It is reasonable to deduce that epigenetic modifications between genomes may contribute to homologous chromosome recognition and cytological diploidization [34].

In most cases, the loss of DNA in allopolyploids is unidirectional, with the elimination of fragments from one parental genome only [35]; incidentally, if applied to *Clonopsis *parthenogens, this would explain why AFLP markers have lost most (but not all) traces of the hybridizations leading to *C. gallica *and *C. soumiae *strains.

However, the main force that drives karyotype diploidization is its higher fitness, because diploidization solves conflicts in the hybrid genome, as well as pairing problems during meiosis. In fact, most of the known diploidized hybrids so far retain meiosis [28-30,33,34]. This seems not to be completely true for the parthenogenetic *C. gallica *and *C. soumiae*: in fact, in *C. gallica *time-scheduled investigations on laid eggs [36] revealed that both European and Moroccan specimens reproduce by apomictic parthenogenesis, albeit of two different kinds, i.e. a meiotic-like mode with two divisions (likely entraining an intra-meiotic structural chromosome doubling) is realized in European specimens of *C. gallica*, while a unique mitosis is required to allow embryo onset in Moroccan *C. gallica*. Like Moroccan *C. gallica*, *C. soumiae *is invariably maturing eggs through a unique mitosis. Therefore, even though some meiotic features are still retained in some strains, most *Clonopsis *make use of mitosis to produce their parthenogenetic progeny. So, at present, a diploid karyotype seems not to be a significant advantage for *Clonopsis*. We can still speculate that all *Clonopsis *hybrids retained some meiosis at the very beginning when they first arose, as happens now in European strains, thus driving fast diploidization, but it seems hard to explain why some strains, once diploidized, soon after lost their meiosis, as should have happened in North African *Clonopsis*. We also tested the specimens for the presence of sex-distorting bacteria, such as Rickettsiales [35], that might have induced parthenogenesis, but we had no evidence for their presence in *Clonopsis *(data not shown).

### Reconstructing *Clonopsis *micro-evolutionary events

As mentioned, pre-meiotic, intra-meiotic or post-meiotic restitution by endomitosis might explain the ploidy increase in *Clonopsis*. To account for this, we may hypothesize that the first step to *Clonopsis *parthenogenetic strains might be a diploid hybrid, showing two complete series of chromosomes of two different species. This hybrid may produce diploid gametes as the outcome of one of the above-mentioned mechanisms of altered meiosis. This may explain how *C. soumiae *originated: in fact, the fusion of two genetically identical diploid hybrid gametes will produce a tetraploid individual, with a diploid-looking karyotype, if the parental chromosomes are different enough. If this is the case, some admixed genome constitution should be easily detectable in *C. soumiae*, which is not always true, judging from some of our AFLP data (Figure 3). On the other hand, *C. gallica *is harder to explain: in fact, if we admit its origin through diploid gametes, its triploid number should be achieved only through the fusion of one haploid and one diploid gamete. In this case, however, some chromosomes will be represented once, the others twice, which seems not to be the case of *C. gallica*. Therefore, we think that the morphologically diploid structure of *Clonopsis *karyotypes ought to be looked for in a different cause.

**Figure 4:**
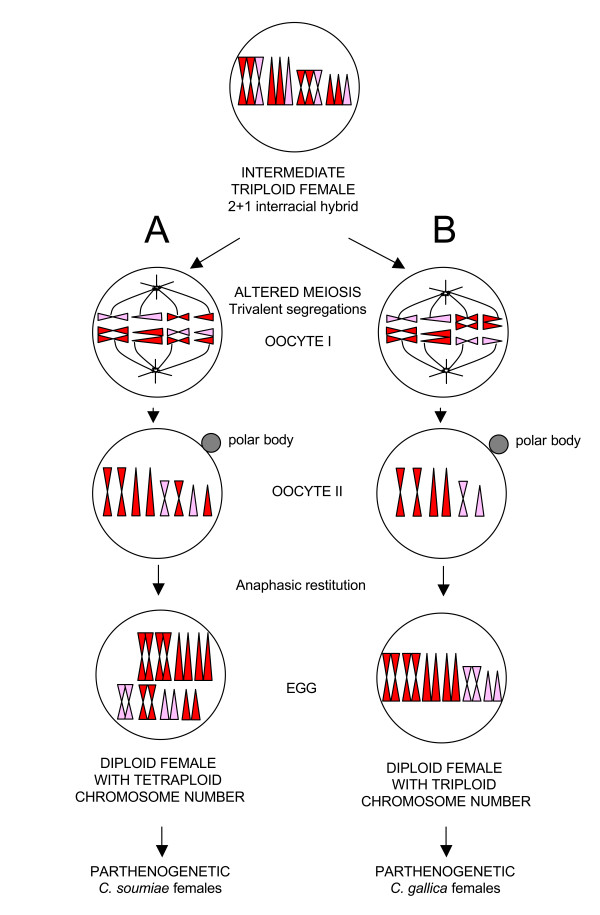
**Diploidization mechanism: origin of parthenogens**. The proposed fast diploidization mechanism through an "intermediate triploid female" with altered meiosis and trivalent segregation. Scheme A: two complete series of chromosomes are segregated to the oocyte II, while the remaining one ends up in the first polocyte. A cytologically normal second meiotic division follows, and an anaphasic restitution (i.e. retention of the polar body) doubles the chromosome number, by the suppression of the second polocyte degeneration. As a result of this process, the oocyte has a tetraploid chromosome number, but chromosomes are in pairs and the karyotype has a perfect diploid look (hypothesis for *C. soumiae *parthenogen origin). Scheme B: half of the trivalents are segregating 2⇔1, the other half 1⇔2. The resulting oocyte II is therefore aneuploid, i.e. half of the chromosomes are present twice, but the other half only once. A normal second division follows, and an anaphasic restitution produces an egg that has a triploid chromosome number, but again the karyotype looks structurally diploid (hypothesis for *C. gallica *strains origin).

Another observation might help in approaching the *Clonopsis *puzzle from another point of view: stick insect eggs are polyspermic and, after fertilization, selective elimination of either egg or sperm nuclei have been recorded in embryo development [37]. Moreover, the presence of more than two haploid sets in the same genome is quite common in stick insects, and triploids are often produced, such as in *Pynackeria *and *Bacillus *taxa (reviewed in [6]). Therefore, we are allowed to hypothesize that a "triploid intermediate female" may be the first step in producing *Clonopsis *polyploid parthenogens. The proposed process is reported in Figure 4.

First, we have to mention that the genome of such hypothetical triploid females might be either derived from both sperm and egg nuclei (by syngamy), or from sperm nuclei only, *via *androgenesis. In androgenesis, the embryo nuclear genome originates from either the doubling of a sperm head or the fusion of two (or more) sperm heads, while the egg pronucleus does not contribute to the genome of the embryo [7]. Androgenesis was first shown in stick insects, and afterwards found in several species of freshwater *Corbicula *clams [38-40], and in the cypress tree *Cupressus dupreziana *[41,42]. At this stage we do not have clear-cut data to assess whether the hypothetical triploid female derived *via *androgenesis or syngamy; however the low genetic differentiation in *Clonopsis *mtDNA might be taken as an indication that parthenogens arose by androgenesis from the same maternal ancestor (i.e. *C. felicitatis*), by keeping its mtDNA and embodying nuclear genomes of extinct or still unsampled paternal bisexuals, as observed in *Pijnackeria *tetraploids [4].

Whether originating from syngamy or androgenesis, the "triploid intermediate female" could be either hybrid or not (i.e. it may have chromosome sets derived from the same or different parental species or races), although hybridity would better explain the alteration of meiosis and parthenogenetic reproduction. In any case, such triploid females must have had problems in correctly segregating chromosomes, since trivalents are expected to occur during meiosis of oocytes I. However, in most cases, chromosome triplets are likely to segregate in a 2⇔1 way, i.e. 2 chromosomes go to one pole and the third goes to the opposite one, a behavior that has been observed many times in triploids [43,44]. This generates a vast array of different chromosome segregations in oocytes II. Two of them are particularly interesting. Case A (Figure 4) segregates two complete series of chromosomes into the oocyte II, while the third one ends up in the first polocyte. A cytologically normal second meiotic division follows, and an anaphasic restitution doubles the chromosome number, likely by the suppression of the second polocyte degeneration (i.e. retention of the polar body). Anaphasic restitution is not new to stick insects, and it has also been found in many other parthenogens [45]. As a result of this process, the new egg gets a tetraploid chromosome number, chromosomes in pairs and the karyotype with a diploid look, while its genome constitution is *de facto *tetraploid. Such an egg will eventually develop into a parthenogenetic female. We suggest that *C. soumiae *strains (*cn *= 72) arose directly in this way. In the second scenario, as reported in scheme B (Figure 4), half of the trivalents are segregating 2⇔1, the other half 1⇔2. The resulting oocyte II is therefore aneuploid, i.e. it has half chromosomes represented twice, while the other half once only. As above, a normal second division follows, and an anaphasic restitution produces an egg that has a triploid chromosome number, but again the karyotype looks diploid and the new genome carries either tetraploid or diploid loci. Eggs like these might have produced *C. gallica *strains (*cn *= 54).

If this is the process that generated *Clonopsis *parthenogenetic strains, it is worth noting that their karyotype diploid-look is just the effect of anaphasic restitution, but their genomes still remain largely (*C. gallica*) or totally (*C. soumiae*) polyploid. This may also account for the partial or complete suppression of meiosis in these strains, because multivalents may occur in such complex multi-copy genomes experiencing large homology regions between non-homologues. As a matter of fact, this is completely different from the above-mentioned diploidization mechanisms, which are processes that restore meiosis after polyploidization.

Unfortunately, sizes and centromere positions are similar for most *Clonopsis *chromosomes and it is not easy to spot whether they are two or fourfold by standard karyotyping, while fine banding techniques that would help in this case have still not been developed for stick insects. Moreover, subsequent minor chromosome changes and/or differences in condensation due to epigenetic modifications of chromatin may further blur chromosome morphology. However, many observations still point to the fact that the proposed process might have happened in *Clonopsis*, since this hypothesis seems to fit very well with the data we have obtained from this molecular analysis. First of all, given that a hypothetical *Clonopsis *triploid hybrid female may segregate 18 trivalents with all possible 2⇔1 segregations, it is interesting to calculate the percentage of eggs with the A or the B constitutions: 7.63x10^-4^% of the eggs will be as in case A, while 18.5% will be like B. Therefore, both events are not unlikely and might have happened many times, because a single *Clonopsis *female lays up to 100 eggs and each population may contain hundreds of females. Moreover, the B segregation scheme is surprisingly common, so that, each time a triploid female does eventually appear, it could easily result in a new *C. gallica *strain. This fits our data, which clearly support a rich polyphyletic origin of *C. gallica *parthenogens. Another surprising observation is well explained by this hypothesis: if both *C. gallica *and *C. soumiae *strains may arise at the same time even from a single triploid female, then they can be largely identical using AFLP loci even if their karyotypes are different, a finding that we observed in the Sefliane (SEF) population and that is hard to explain with other hypotheses.

We may recall that the full suppression of meiosis in *Clonopsis *parthenogens may have occurred at a later stage, also because the hypothetical triploid *Clonopsis *female was possibly a hybrid between races or subspecies, thus having homoeologues in its genome. It is worth noting that homoeologues are not easily detected by AFLP markers, because they mainly differ in repetitive DNA content (i.e. copy number). This could explain why *Clonopsis *unisexuals do appear, either admixed or not, depending on the overall nucleotide divergence of the homoeologues carried by each "intermediate triploid female" that gave rise to the strains.

It still remains debatable how, among many different combinations resulting from trivalent differential segregation patterns, only *cn *= 54 and *cn *= 72 *Clonopsis *parthenogenetic strains survived: we may suggest that some sort of genome balancing is implied here, which made *cn *= 54 and 72 strains able to survive better as parthenogens. Moreover, specific epigenetic genome silencing might also be conceivable in balancing the hybrid genome made up of 54 and 72 chromosomes. The complete lack of data on genetic and epigenetic characterizations of phasmids prevents us from addressing this point better.

**Figure 5:**
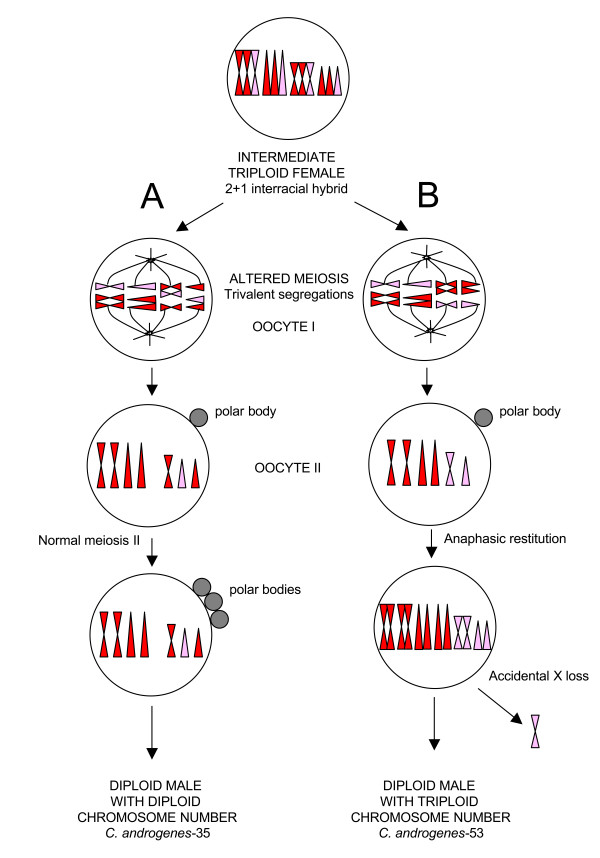
**Diploidization mechanism: origin of *C. androgenes***. The proposed fast diploidization mechanism through an "intermediate triploid female" with altered meiosis and trivalent segregation. Scheme A: *cn *= 35 androgen might have appeared or by direct hybridization of species/forms, or by incorrect X segregation during the meiosis I of the triploid female. Both ways might give rise to a male with *cn *= 35, with a numerically and structurally diploid karyotype, but with a suppressed meiosis, an indication of a possible hybrid origin. Scheme B: *cn *= 53 androgen would derive directly from a *C. gallica-*like strain: the accidental loss on an X chromosome produced a male individual with suppressed meiosis, which started the *cn *= 53 androgenetic clone, with numerically triploid karyotype, but with diploid structure.

Following our hypothesis, we might also speculate on how androgens may have originated. It is quite well known that, even within all-female diploid strains, stick insect males (which are X0) may arise through accidental loss of one sex chromosome, as observed in *Bacillus *[7]. An accidental sex chromosome loss could well explain the *cn *= 53 androgen, which would therefore derive directly from a *C. gallica *strain: actually, following the B scheme in Figure 5, we may suppose that the accidental loss of an X chromosome produced a male individual with suppressed meiosis, which started the *cn *= 53 androgenetic clone. On the other hand, the case of the *cn *= 35 male is harder to explain. First of all, it must be noted that AFLP loci exclude its direct derivation from *C. felicitatis*; moreover its suppressed meiosis, in spite of a diploid chromosome constitution, may be an indication of a hybrid origin. Actually, we may speculate that such males might have appeared in two different ways, i.e. by direct hybridization of species/forms leading to a diploid hybrid male, or by incorrect X segregation during the meiosis I of the "intermediate triploid female" of the A scheme, as depicted in Figure 5. Both ways might give rise to a diploid male with *cn *= 35 with suppressed meiosis because of its hybrid constitution. It is evident that, although some differences do exist, both *cn *= 35 and 53 males share most of their AFLP alleles, a case that strongly recalls Sefliane polyploid females and which may indicate that both arose from a single "triploid intermediate female", likely within a common process such as the one proposed above.

## Conclusions

In conclusion, we here suggest that *C. gallica *and *C. soumiae *parthenogens might have arisen many times through "intermediate triploid females" with altered meiosis. The mechanism proposed here explains all the data we have obtained so far, and, quite significantly, its simple process does not require any new *ad-hoc *cytological mechanism, since all the proposed steps have been observed many times in closely related stick insects. Given the complexity of *Clonopsis *micro-evolutionary history, the detailed events leading to each strain are not easy to disentangle at the moment, and this would require larger collecting campaigns in Northern Africa and Europe. Moreover, a genome characterization of *Clonopsis*, which unfortunately is not available, would better support our hypothesis, and a fine FISH hybridizing technique, such as "chromosome painting", would also help in supporting the proposed model. Our micro-evolutionary scenario for *Clonopsis *needs more in-depth analyses to be tried out, nevertheless we feel that this is a sound "working hypothesis" that merits further study.

## Methods

### Sample collection

Figure 1 shows collection localities, with population acronyms for the new Moroccan samples, and also reports additional locations in Portugal, Spain and Italy from which some reference *C. gallica *specimens were utilized. North African forms are particularly hard to collect in a pretty unsafe area such as Northern Algeria and the Rif area of Morocco. At any rate, in 2006, we were able to collect in the Rif area, which is probably the spreading centre of *Clonopsis*. North African sample included Moroccan *C. gallica*, the bisexual *C. felicitatis *(*cn *= 36/35, XX/X0), the all-female *C. soumiae *(*cn *= *72*), two strains of ameiotic males, *C. androgenes-35 *and *C. androgenes-53*, with *cn *= 35 (X0) or 53 (X0), and a single *C. maroccana *(*cn *= 22/21, XX/X0) female, used as outgroup when needed (see below). They were characterized for mitochondrial gene cytochrome oxidase subunit 2 (*cox2*, partial sequence). North African populations were also analyzed using AFLP. Detailed data are reported in Table 1.

### Mitochondrial DNA analysis

Total genomic DNA was extracted using the DNeasy Blood & Tissue Kit (Qiagen). Partial sequence of *cox2 *was amplified and directly sequenced according to [19]. The primers utilized were TL2-J-3034 and TK-N-3785 [46]. Sequencing covered 639 bp coding for 213 aminoacids of the Cytochrome Oxidase subunit 2 and corresponds to the gene region sequenced in several insect orders [47]. All sequences were aligned with the Clustal algorithm of MEGA 3.1 [48]. A Bayesian analysis was performed using MrBayes 3.1 (20,000,000 generations; [49]) (Figure 2A). A Templeton's network [50] was obtained with TCS 1.21 [51] (Figure 2B). *Bacillus grandii grandii*, *Bacillus atticus atticus *and *Bacillus rossius redtenbacheri *were utilized as outgroups in phylogenetic reconstructions based on *cox2*.

### AFLP markers

DNA fingerprinting with selectively neutral AFLP markers was produced according to [52], using EcoRI/TaqI as restriction enzymes. After a preliminary screening, three highly polymorphic primer pairs carrying ACA/AAC, ACA/AAG and ATG/AAC as selective nucleotides were assayed on 100 ng of total genomic DNA. AFLP fragments were separated by electrophoresis on 8% polyacrylamide gels and the polymorphisms were visually scored as dominant markers, coding with 1 the presence and with 0 the absence of the band. Markers with more than 5% of missing data were removed from the definitive dataset.

The genetic relationships at individual level were assessed by the Factorial Correspondence Analysis (FCA), a statistical method enabling analysis and description graphically and synthetically of two-way or multi-way contingency tables. For this, the software GENETIX 4.05 was used [53] (see additional file 1).

To assess the molecular structure of Moroccan *Clonopsis *we used a model-based bayesian procedure as implemented in the software STRUCTURE 2.2 [54,55]. This model enables identification of the K (unknown) populations of individuals, and the probabilistic assignment of each individual to one or several populations if its genotype indicates that it is admixed. The model assumes that the loci are unlinked and at linkage equilibrium. STRUCTURE version 2.2 calculates a logarithmic probability for the data being assigned to a given number of clusters, based on minimizing linkage between clusters, and maximizing linkage within.

A Minimum Evolution tree (ME, Figure 3A) based on AFLP markers was calculated using PAUP 4.0 [56]; support for each node was obtained using bootstrap (1000 replicates, [57]). Given the results of *cox2, C. maroccana *has been used as an outgroup in the AFLP-based tree, since more phylogenetically distant species (such as *Bacillus *ssp.) would not be easily comparable using AFLP markers, which are more suitable for genetic analyses of population strains or closely related species [20], and have been proved to be resolving for phylogenetic structure in rapidly evolving systems [19].

## Authors' contributions

LM and FG carried out *cox2 *mitochondrial analysis, sequence alignment, statistical analysis and helped to draft the manuscript. MPE carried out AFLP analysis and helped to draf the manuscript. VS collected the specimens and participated in the design of the study. MPA conceived the study, participated in its design and coordination and drafted the manuscript. All authors have read and approved the final manuscript.

## Supplementary Material

Additional file 1 **Factorial Correspondence Analysis**. The genetic relationships at individual level were assessed by Factorial Correspondence Analysis (FCA), using the GENETIX software. Acronyms as in Table 1. Firstly, FCA analysis was run on the complete matrix of AFLP data. The three-dimensional graph (not shown) highlights the fact that the presence of TAR6 - i.e. *C. maroccana *- "pushes" the other individuals to the top of the chart, as it assumes an outgroup behavior, thus confirming that *C. maroccana *is genetically very different from the other specimens included in the study. Therefore, we ran FCA excluding *C. maroccana *from the dataset to better trace relationships between the remaining specimens. The graph obtained is reported here. As expected, this time the individuals occupy three-dimensional space in a more homogeneous way. In more detail, amphigonic *Clonopsis *from Tetouan (TET) form a large cloud at one pole, while the parthenogenetic females of Targuist (TAR) and Sefiane (SEF) are at the opposite one, along the first axis (25.3% of the total inertia); finally, the remaining individuals spread over the intermediate space between these two extremes, almost in seamless continuity. In addition, the two androgenetic males with 53 chromosomes from Targuist (TAR43 and TAR47) are separated from the other individuals along axis 2 (10.65% of the total inertia), while the third axis (7.42%) tends to split two amphigonic males from Tetouan (TET56 and TET57) from the group, together with the parthenogenetic females *cn *= 54 from Oued Laou (OLA). It can also be noted that TET, OLA, and TAF samples form coherent groups, while TAR and SEF specimens are grouped in several subsets, thus indicating that some different genetic entities were sampled in the same localities. Click here for file
