# Supplementary material for: Reticulate evolution in stick insects: the case of Clonopsis (Insecta Phasmida)
Source: BMC Evol Biol. 2010 Aug 25;10:258. doi: 10.1186/1471-2148-10-258 (PMC2936309; doi:10.1186/1471-2148-10-258)
Supplement: Additional file 1 — Factorial Correspondence Analysis. The genetic relationships at individual level were assessed by Factorial Correspondence Analysis (FCA), using the GENETIX software. Acronyms as in Table 1. Firstly, FCA analysis was run on the complete matrix of AFLP data. The three-dimensional graph (not shown) highlights the fact that the presence of TAR6 - i.e. C. maroccana - "pushes" the other individuals to the top of the chart, as it assumes an outgroup behavior, thus confirming that C. maroccana is genetically very different from the other specimens included in the study. Therefore, we ran FCA excluding C. maroccana from the dataset to better trace relationships between the remaining specimens. The graph obtained is reported here. As expected, this time the individuals occupy three-dimensional space in a more homogeneous way. In more detail, amphigonic Clonopsis from Tetouan (TET) form a large cloud at one pole, while the parthenogenetic females of Targuist (TAR) and Sefiane (SEF) are at the opposite one, along the first axis (25.3% of the total inertia); finally, the remaining individuals spread over the intermediate space between these two extremes, almost in seamless continuity. In addition, the two androgenetic males with 53 chromosomes from Targuist (TAR43 and TAR47) are separated from the other individuals along axis 2 (10.65% of the total inertia), while the third axis (7.42%) tends to split two amphigonic males from Tetouan (TET56 and TET57) from the group, together with the parthenogenetic females cn = 54 from Oued Laou (OLA). It can also be noted that TET, OLA, and TAF samples form coherent groups, while TAR and SEF specimens are grouped in several subsets, thus indicating that some different genetic entities were sampled in the same localities. [file 1471-2148-10-258-S1.DOC]

## Additional file 1


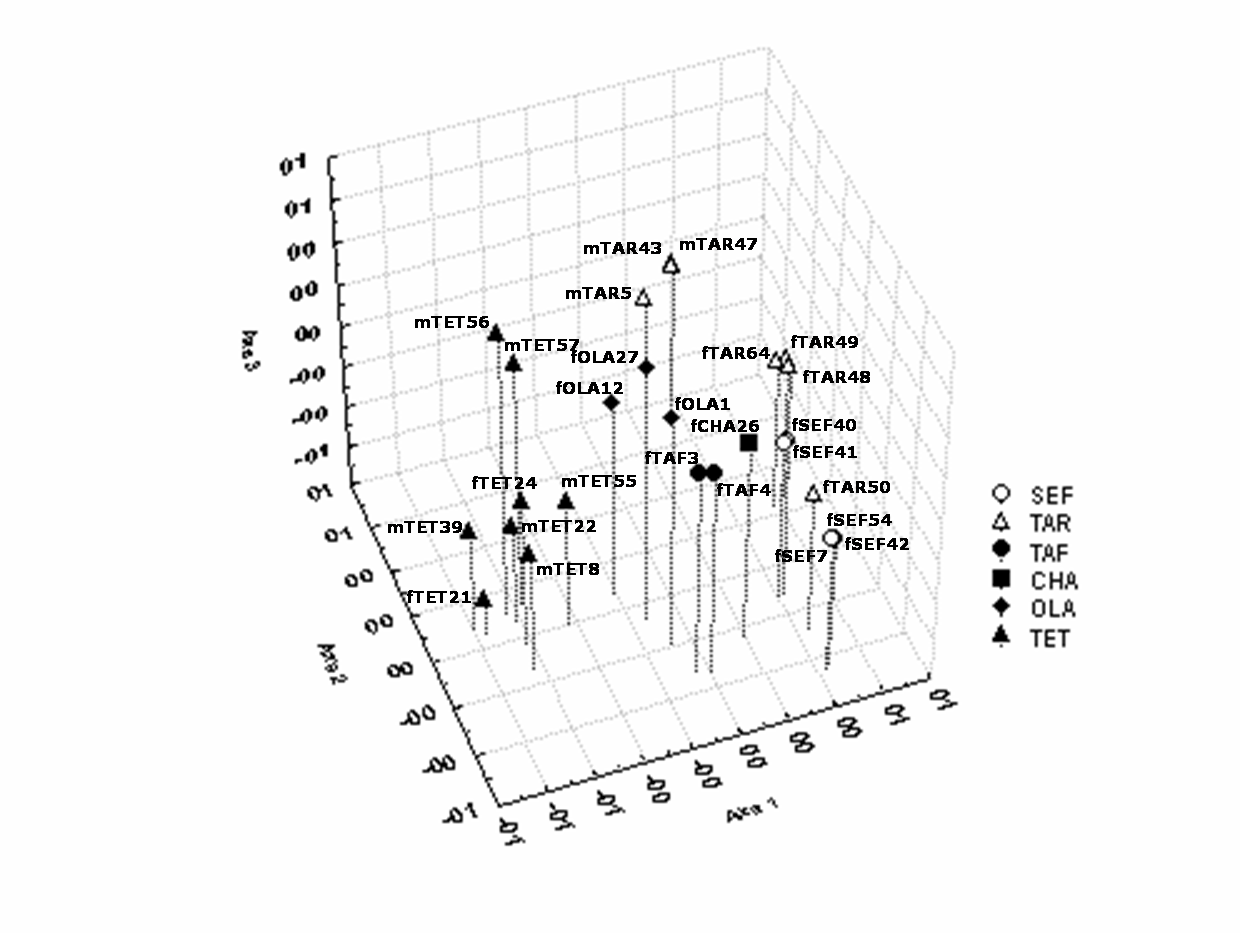


**Factorial Correspondence Analysis**

The genetic relationships at individual level were assessed by Factorial Correspondence Analysis (FCA), using the GENETIX software. Acronyms as in Table 1. Firstly, FCA analysis was run on the complete matrix of AFLP data. The three-dimensional graph (not shown) highlights the fact that the presence of TAR6 – i.e. *C. maroccana* – “pushes” the other individuals to the top of the chart, as it assumes an outgroup behavior, thus confirming that *C. maroccana* is genetically very different from the other specimens included in the study. Therefore, we ran FCA excluding *C. maroccana* from the dataset to better trace relationships between the remaining specimens. The graph obtained is reported here. As expected, this time the individuals occupy three-dimensional space in a more homogeneous way. In more detail, amphigonic *Clonopsis* from Tetouan (TET) form a large cloud at one pole, while the parthenogenetic females of Targuist (TAR) and Sefiane (SEF) are at the opposite one, along the first axis (25.3% of the total inertia); finally, the remaining individuals spread over the intermediate space between these two extremes, almost in seamless continuity. In addition, the two androgenetic males with 53 chromosomes from Targuist (TAR43 and TAR47) are separated from the other individuals along axis 2 (10.65% of the total inertia), while the third axis (7.42%) tends to split two amphigonic males from Tetouan (TET56 and TET57) from the group, together with the parthenogenetic females *cn*=54 from Oued Laou (OLA). It can also be noted that TET, OLA, and TAF samples form coherent groups, while TAR and SEF specimens are grouped in several subsets, thus indicating that some different genetic entities were sampled in the same localities.
